# Impact of Omega-3 Fatty Acids on Cognitive Outcomes in Children With Autism Spectrum Disorder: A Systematic Review

**DOI:** 10.7759/cureus.80291

**Published:** 2025-03-09

**Authors:** Bushra Sumra, Cyril Kocherry, Hina Shamim, Kiran Jhakri, Moath Al-Shudifat, Lubna Mohammed

**Affiliations:** 1 Clinical Research, University of London, London, GBR; 2 School of Medicine, Ninewells Hospital, Dundee, GBR; 3 Pediatrics, Baqai Medical University, Karachi, PAK; 4 Internal Medicine, Shahjalal University of Science and Technology, Sylhet, BGD; 5 Internal Medicine, Faculty of Medicine, Cairo University, Cairo, EGY; 6 Internal Medicine, Dr. VRK Women's Medical College, Hyderabad, IND

**Keywords:** autism, autism spectrum disorder (asd), developmental pediatrics, docosahexaenoic acid (dha), eicosapentaenoic acid (epa), gut-brain connection, omega-3 fatty acids, pediatric neuroinflammation

## Abstract

Autism spectrum disorder (ASD) is defined as a complex neurodevelopmental disorder that is characterized by a set of deficits not limited to social communication, which is restricted and repetitive behaviors. The prevalence of autism has been seen to be consistently increasing globally. Autism is multifactorial in its etiology, and it involves several physiological systems, including the central nervous system and the gut-brain axis. Omega-3 fatty acids are essential for neural development and functionality, particularly eicosapentaenoic acid (EPA) and docosahexaenoic acid (DHA). They both play a crucial role in not only reducing the neuroinflammation associated with autism but also supporting cognitive processing as well. Given the low levels of omega-3 noted in ASD individuals, this systematic review aims to assess the influence of omega-3 supplementation on cognitive outcomes in children with ASD.

The systematic review was done following the Preferred Reporting Items for Systematic Reviews and Meta-Analyses (PRISMA) guidelines, where different databases were assessed across PubMed, Science Direct, Cochrane Library, Google Scholar, and Scopus. MeSH terms used included keywords "Omega-3", "EPA", "DHA" AND "Autism Spectrum Disorder" OR "ASD". Articles published between 2007 and 2023 that focused on ages 2 to 18 years were screened, and cognitive outcomes relevant to omega-3 supplementation were included. Studies with inadequate access to full text excluded non-human trials and older individuals. After generating 25,312 articles, 211 were selected for further review, with 11 meeting the inclusion criteria.

The articles reviewed panned over five different countries that involved omega-3 supplementation lasting up to one year. Results suggested that DHA and EPA supplementation may improve cognitive functions such as memory, attention, and executive functioning in children with ASD. The prefrontal cortex development was associated with DHA supplementation, whereas EPA showed improved emotional regulation and reduced neuroinflammation. However, conclusive results were not reached as there was variability in study designs, different dosages, and assessment methods. The power of the studies conducted was also noted to be limited. While promising, extensive research and trials are required to standardize the dosage of omega-3 and the length of intervention. Future studies should aim to identify the long-term effects of omega-3 supplementation, understand the gut-brain axis, and investigate the combination of omega-3 with other therapies to improve cognitive functioning.

## Introduction and background

Autism spectrum disorder (ASD) refers to a neurodevelopmental disorder that is identified by deficits in socio-emotional reciprocity; it ranges from abnormal social approaches and inadequacy of neurotypical back-and-forth communication to minimization of shared interests, emotions, and affect and response to a total lack of initiation of social interaction [1]. The prevalence of autism has increased greatly in developed countries to as high as 1.5% of the population [2] and is measured to be approximately 2.4% in the United States [3]. The WHO estimates the worldwide prevalence to be 1 in 160 children with autism. Autism is thought to involve various systems, including but not limited to the peripheral nervous system, which has an important part to play when addressing sensory-neural pathways, especially when we are looking at sensory processing disorders, central nervous systems, the musculoskeletal system, and fairly recently the digestive system, which has now formulated the brain-gut axis [4]. It is still not clear where the exact etiology of autism arises from, and the pathogenesis is also not well understood. However, the evidence shows that it has multiple systems playing a role and leading to multiple comorbidities, which finally flare and show the core symptoms present in ASD.

Emerging evidence suggests that the gut microbiome plays a crucial role in neurodevelopment and may be significantly altered in children with ASD [5]. The gut-brain axis, a bidirectional communication pathway between the gut and the central nervous system, has been implicated in ASD pathophysiology [6]. Omega-3 fatty acids, particularly DHA and EPA, have been found to modulate gut microbiota composition, reduce intestinal inflammation, and enhance gut barrier integrity [7,8]. These mechanisms suggest that omega-3 supplementation could exert cognitive benefits through direct neural mechanisms and the gut-brain axis, potentially alleviating some ASD-related symptoms.

Omega-3 fatty acids, such as eicosapentaenoic acid (EPA) and docosahexaenoic acid (DHA), are deemed crucial for the development of the prefrontal cortex, hippocampus, and cerebral cortex, as well as the amygdala and hypothalamus, respectively [9]. Omega-3 fatty acids are a crucial part of neural membranes and serve to promote neurotransmitter function and reduction of neuroinflammation. DHA has been, in particular, linked to supporting memory and cognitive flexibility. These fatty acids are thereby referred to as essential fatty acids, as humans are incapable of synthesizing them and are supplemented via diet or deep-sea fish oil [10]. Approximately 60% of patients on the autism spectrum are deduced to have immune dysfunction; this gives a direct link to essential fatty acids and neuroinflammation [11]. Lower levels of omega-3 in the blood of ASD individuals were linked with an overproduction of pro-inflammatory cytokines [12]. The reason why these dietary insufficiencies are present in patients with autism can be linked to the behavioral nature, as they tend to be picky eaters; this has overwhelmingly led to autoantibodies, which have damaged neural and glial molecules and therefore led to omega-deficient disorders, as well as increased inflammatory cytokines and oxidative stress, which can finally be attributed to the manifestation of the symptoms evident [13].

Comprehending the possible advantages of these nutrients in light of the neurodiversity of ASD is essential. The goal of this systematic review is to compile the vast body of research on how omega-3 supplements affect cognitive outcomes in kids on the autism spectrum.

## Review

Materials and methods

**Figure 1:**
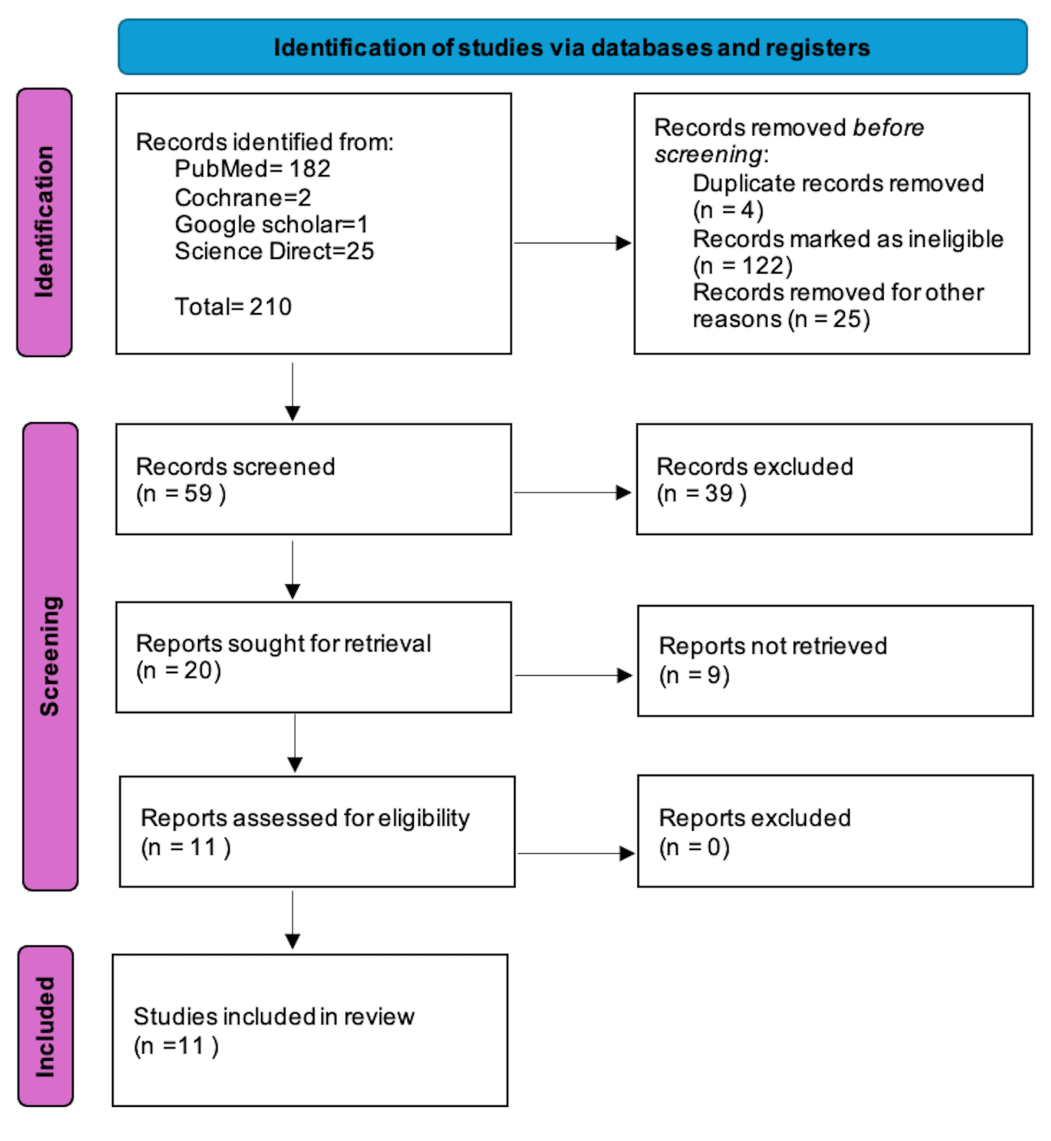
PRISMA flow diagram showing the process of article selection PRISMA: Preferred Reporting Items for Systematic Reviews and Meta-Analyses

To ensure transparency in the review process, search criteria were conducted in accordance with the guidelines presented in the Preferred Reporting Items for Systematic Reviews (PRISMA 2020). Duplicate articles were removed using EndNote, and the titles and abstracts were independently screened. This involved a systematic search across PubMed, Cochrane Library, Science Direct, Google Scholar, and Scopus, among other databases. MESH terms and combinations were limited to "Omega-3 Fatty Acids" OR "EPA" OR "DHA" OR "Omega" AND "Autism Spectrum" OR "ASD" OR "Autism Spectrum Disorder" OR "Autism" (Table 1). A total of 25,312 articles were screened, and after the inclusion and exclusion criteria were implemented, only 210 articles were found to be eligible for further screening (Figure 1).

**Table 1:** Terminology and MeSH words

Terminology	MeSH words
Autism	Autism OR( "Autism Spectrum Disorder/classification"[Majr] OR "Autism Spectrum Disorder/diet therapy"[Majr] OR "Autism Spectrum Disorder/epidemiology"[Majr] OR "Autism Spectrum Disorder/metabolism"[Majr] OR "Autism Spectrum Disorder/microbiology"[Majr] OR "Autism Spectrum Disorder/physiopathology"[Majr] )
Omega-3	Omega-3 OR ( "Fatty Acids, Omega-3/administration and dosage"[Mesh] OR "Fatty Acids, Omega-3/adverse effects"[Mesh] OR "Fatty Acids, Omega-3/blood"[Mesh] OR "Fatty Acids, Omega-3/deficiency"[Mesh] OR "Fatty Acids, Omega-3/genetics"[Mesh] OR "Fatty Acids, Omega-3/metabolism"[Mesh] OR "Fatty Acids, Omega-3/pharmacokinetics"[Mesh] OR "Fatty Acids, Omega-3/physiology"[Mesh] OR "Fatty Acids, Omega-3/therapeutic use"[Mesh] OR "Fatty Acids, Omega-3/toxicity"[Mesh] )
Cognitive	Cognitive OR ( "Neuropsychological Tests/history"[Majr] OR "Neuropsychological Tests/standards"[Majr] OR "Neuropsychological Tests/statistics and numerical data"[Majr] )

Inclusion and exclusion criteria

For the final study reviews, a set of criteria was employed, which included the articles that offered unrestricted access to their full text for evaluation. It was also restricted to articles in the English language between the years 2007 and 2023. It focused on both genders but restricted the age between 2 years and 18 years. It focused on articles that used omega-3 interventions and that had cognitive outcomes assessed using primary tools such as attention, memory, or executive functioning. Studies published in different languages before 2015 or involving ages less than two years and more than 18 years, as well as animal studies, meta-analyses, and conference abstracts without original data, were considered ineligible. Table 1 shows the MeSH terms used for this study.

Quality check and data extraction

Two independent researchers assessed the quality of the topics selected. They organized the studies by first author name, year of publication, origin country, omega-3 concentrations, and outcomes, as shown in Table 2 below.

**Table 2:** Summary of cognitive outcomes from selected studies on omega-3 supplementation in children with autism spectrum disorder

Study (author, year)	Country of origin	Omega-3 concentration (dose)	Cognitive outcomes (key findings)	Quality rating (e.g., Newcastle-Ottawa Scale)
Bent et al., 2009 [14]	USA	1.3 g	Improvement in social communication, no significant effect on hyperactivity	High
Bent et al., 2011 [15]	USA	1.3 g	Significant improvement in executive function and communication	High
Agostoni et al., 2017 [16]	Italy	345 mg	Moderate improvement in attention and executive function	Moderate
Jiang et al., 2023 [17]	China	1.3-1.5 g	Improved memory retention, a slight improvement in attention	High
James et al., 2011 [18]	USA	NA	Mixed results in executive functioning and social responsiveness	Moderate
Veselinović et al., 2021 [19]	Serbia	200 mg	Reduced neuroinflammation, improved emotional regulation	High
Chang and Su, 2020 [20]	China	>750 mg	Improvement in attention span, minor effects on mood	Moderate
Sathe et al., 2017 [21]	USA	250 mg	Slight improvement in social interaction, a limited effect on cognition	Moderate
Horvath et al., 2017 [22]	USA	250 mg	Minimal effect on cognitive measures, moderate improvement in behavior	Low
Cheng et al., 2017 [23]	Taiwan	200 mg	Reduction in stereotypy, moderate increase in attention	Moderate
Bozzatello et al., 2021 [24]	USA	750 g	Increase in memory recall, some improvement in cognitive flexibility	High

For randomized controlled trials (RCTs), the Cochrane Risk of Bias Tool was used and summarized in Table 3. There was no suggestion of risk of bias in three studies, with two presenting a moderate overall risk resulting from blinding, attrition, and other biases.

**Table 3:** Cochrane Risk of Bias

Study (author, year)	Randomization	Blinding	Attrition bias	Reporting bias	Other bias	Overall risk
Bent et al., 2009 [14]	Low	Low	Low	Low	Low	Low
Agostoni et al., 2017 [16]	Low	High	Moderate	Low	Moderate	Moderate
Jiang et al., 2023 [17]	Low	Low	Low	Low	Low	Low
Veselinović et al., 2021 [19]	Low	Low	Low	Low	Low	Low
Sathe et al., 2017 [21]	Low	Moderate	Moderate	Low	Moderate	Moderate

Discussion

A total of 11 papers were assessed from five countries. All the subjects in this study were noted to be between 2 and 18 years old, and the omega-3 intervention lasted no more than one year. The DSM criteria were adapted to diagnose ASD [1,2].

There have been no breakthroughs in the treatment of ASD in the pharmaceutical world, and children on the spectrum are considered picky eaters [2]. As a result, nutritional therapy is one of the fields that is being looked into to address the nutritional deficiencies and alleviation of the core symptoms in ASD [3]. Eleven studies analyzing the effects of supplementing omega-3 had variable results.

The results from the systematic review show that omega-3 fatty acid supplementation, particularly DHA and EPA, had potential benefits for children on the spectrum. DHA supplementation was shown to improve prefrontal cortex development and showed improvement in memory, attention, and cognitive flexibility, whereas EPA supplementation showed better regulation of emotional responses by reducing neuroinflammation [10,18,19]. A study by Horvath et al. [22] included other functional parameters, including communication and social interaction.

Cognitive improvements shown with DHA and EPA supplementation included the following:

Improved Memory and Learning

Jiang et al. [17] reported significant improvements in memory retention, particularly in short-term memory recall. This aligns with DHA’s role in hippocampal development. Bozzatello et al. [24] showed improved memory recall and heightened cognitive abilities with DHA supplementation over a six-month period.

Attention and Focus Improvement

Chang and Su [20] found sustained attention in children supplemented with doses of >750 mg, and Agostoni et al. [16] found moderate improvement in tasks involving sustained attention.

Executive Functioning

Bent et al. [14] observed improved executive functioning and communication, which further stratified the role of EPA in the frontal cortex, and Veselinović et al. [19] highlighted completion and problem-solving abilities improvements.

Emotional Regulation

EPA supplementation showed improved emotional regulation and reduced impulsivity and aggression [18,19].

Social Communication

Bent et al. and Horvath et al. [15,22] reported slight social interaction and communication improvements. These suggest cognitive benefits that extend from supplementing omega-3 fatty acids.

According to the results of this systematic review, children with ASD are proposed to improve cognitively by taking supplements of omega-3 fatty acids, especially EPA and DHA. Improvements in executive functioning, memory, attention, and emotional control were noted in several trials [14,23,24]. These enhancements are consistent with the mounting evidence that omega-3 fatty acids benefit the growth and health of the brain. These findings offer crucial insights into the potential for nutritional interventions to improve cognitive outcomes, especially in light of the complicated neurodevelopmental profile of children with ASD [25,26].

Omega-3 fatty acids play a vital role in reducing neuroinflammation; this is possibly what exhibits some of the symptomatology of ASD. Many children on the spectrum were noted to have elevated levels of pro-inflammatory cytokines and increased oxidative stress, which impair optimal brain functioning, disrupt neurotransmission, and cause cognitive hindrance [9,12]. DHA and EPA have both been shown to improve neuronal function, lower inflammation, and balance neurotransmitters to help control the immunological response in the brain [15,18]. In addition, DHA’s presence in the hippocampus promotes improved learning outcomes by assisting in memory formation [20]. Several studies also highlight improved abilities to retrieve and retain memories, especially with supplementation lasting over six months [21].

EPA, on the other hand, was noted to cause better emotional regulation, which ultimately helps reduce meltdowns, impulsivity, and aggression, including self-harm [16,17]. They also boost cognitive functioning, as an emotional outburst does not fare well with better cognition. The stability provided in such scenarios supports better cognitive outcomes through improved task completion, self-regulation, and problem-solving abilities [20].

Limitations

Despite promising findings, the results of this review are limited by differences in study designs, sample sizes, and assessment methods. Differences in omega-3 dosages, intervention durations, and cognitive evaluation tools limit the generalizability of the results. Furthermore, the possibility of publication bias and metabolic variability among children with ASD makes it difficult to reach firm conclusions about the efficacy of omega-3 supplementation. Larger, well-controlled studies using standardized methodologies are required to confirm these findings.

Recommendations

Future research should focus on standardizing omega-3 dosage and intervention durations to determine the best levels for cognitive improvement in children with ASD. Finding the ideal dose-response connection is made more difficult by the variation in omega-3 dosages utilized in different investigations. While some research found that doses as low as 200 mg/day improved cognitive function, other studies found that doses greater than 750 mg/day were necessary to show meaningful effects. This raises the possibility of a threshold effect, in which greater dosages may have more noticeable cognitive and behavioral effects, whereas lower amounts may offer only modest neuroprotection. However, negative consequences, including gastrointestinal distress or altered immunological responses, can also result from consuming too much omega-3 [27]. Optimizing omega-3’s therapeutic benefits in populations with ASD requires large-scale research to provide a uniform dose approach.

Adopting more uniform cognitive assessment tools to facilitate cross-study comparisons and strengthen the evidence base is also crucial. Behavioral observations, parent-reported questionnaires, and various neuropsychological tests were used in the studies; each had its own inherent biases and sensitivities. Standardized neuropsychological tests, like those employed by Agostoni et al. [16], offer more objective measurements of cognitive function, while parent-reported gains, like those reported by Chang and Su [20], may be prone to subjective interpretation or placebo effects. Cross-study comparisons are further restricted by the absence of a generally recognized cognitive evaluation methodology for ASD therapies [28].

Small sample sizes in many studies are another important drawback that limits how far the results can be applied. To confirm the effectiveness of omega-3 supplementation in a variety of populations with ASD, larger and more thorough trials are required [28]. Additionally, the range of intervention durations raises the possibility that longer-term supplementation could produce more reliable outcomes [29-31].

Future directions

Additional research should look into the long-term effects of omega-3 supplementation on neurodevelopmental outcomes. Furthermore, studying the synergistic effects of omega-3 with other nutritional interventions, such as vitamin D or probiotics, may provide a more complete picture of its role in ASD management. Longitudinal studies with larger sample sizes will help to validate omega-3’s cognitive benefits in ASD patients.

Participant stratification underlies all future work, as little is currently known about individual metabolic variations and baseline omega-3 status in ASD populations. While some children may already have adequate dietary consumption, limiting extra benefits, others may naturally have lower omega-3 levels, making them more susceptible to supplements. Baseline omega-3 levels and individual metabolic variations are key factors affecting the effectiveness of omega-3 supplementation [32]. Variations in fatty acid metabolism would intuitively affect the potential cognitive benefits of omega-3 supplementation [33]. The efficiency with which people transform dietary omega-3 precursors into bioactive forms, for instance, may be impacted by differences in the FADS2 gene, which controls fatty acid metabolism [34], supporting the argument for metabolic and genetic screening.

## Conclusions

This systematic analysis shows that omega-3 fatty acid supplementation may improve cognitive outcomes in children with ASD, namely executive functioning, memory, and attention. While the overall data indicate possible advantages, the variety in trial designs, doses, and assessment methodologies restricts the capacity to make firm conclusions. Nonetheless, the overall tendency favors the use of omega-3 fatty acids as a supplementary intervention to improve cognitive performance in ASD.

The long-term effects of omega-3 supplementation in ASD are still unclear as brain development continues into adulthood. While it may support cognitive and behavioral improvements, there is no strong evidence that early supplementation leads to lasting benefits. Future research should explore how omega-3 works with other nutrients like vitamin D and zinc and conduct larger studies to understand its long-term impact.
